# Role of Aspartate 42 and Histidine 79 in the aiPLA2 activity and oligomeric status of Prdx6 at low pH

**DOI:** 10.21203/rs.3.rs-5129146/v1

**Published:** 2024-12-23

**Authors:** Pushpa Kakchingtabam, Sharifun Shahnaj, Anju Kumari, Japani Longjam, A. N. Wungnaopam, Khundrakpam Herojit, Rajendrakumar Singh Laishram, Aron B. Fisher, Hamidur Rahaman

**Affiliations:** Manipur University; Manipur University; University of Delhi; Manipur University; Manipur University; Manipur University; University of Delhi; University of Pennsylvania Perelman School of Medicine; Manipur University

**Keywords:** peroxiredoxin 6, calcium independent phospholipase A2, 1, 2,-bis palmitoyl-sn-glycero-3-phosphocholine, circular dichroism, thioredoxin, π glutathione S-transferase

## Abstract

Peroxiredoxin 6 (Prdx6), a unique non-seleno peroxidase, is a bifunctional protein with GSH peroxidase at pH 7.4 and calcium independent phospholipase A_2_ (aiPLA_2_) activities at pH 4.0. Changes in pH brings about alteration in the conformational and thermodynamic stability of Prdx6. For instance, under acidic condition (pH 4.0), Prdx6 forms higher oligomers with concommittant gain in aiPLA_2_ activity that is resistant to thermal denaturation. However, there has been no molecular level understanding of how low pH induces formation of oligomers. In the present study, site directed mutagenesis of two conserved amino acid residues, Asp42 and His79, was used to study the molecular basis for the influence of pH on the oligomeric state of Prdx6. We observed that mutation at Asp42 and His79 residues by Ala did not result in a significant change in its peroxidase activity at neutral pH 7.4 but its aiPLA_2_ activity at low pH 4.0 decreased significantly. At this pH condition, both mutants exhibit highly conserved alpha-helix content but fluctuating tryptophan micro-environment with partly exposed hydrophobic patches that renders the formation of oligomers. DLS measurements and analytical SEC revealed that Wt Prdx6 forms oligomers at low pH but not the mutan proteins suggesting the importance of these residues in pH sensing and oligomerization. These results suggest that Asp42 and His79 interact each other to induce conformational change of Prdx6 that triggers the oligomerization of Prdx6 at low pH.

## Introduction

Peroxiredoxins (Prdx) are a family of antioxidant enzymes that play a crucial role in protecting cells from oxidative stress by detoxifying reactive oxygen species (ROS). Prdx proteins are ancestral thiol-dependent selenium- and heme-free peroxidases involved in the detoxification of 90% of cellular peroxides including hydrogen peroxide, peroxinitrite and hydroperoxides due to its high abundance in a wide range of cells and its high catalytic efficiencies in comparison to other peroxidases such as catalase, horseradish peroxidase (HRP) and glutathione peroxidase (GPx) etc.^1–6^; thus, Prdxs help to protect cellular components such as other proteins, lipids, and DNA from oxidative damage.

Prdxs have been categorized broadly into different isoforms (Prdx1–6) based on their structural characteristics and specific functions ^2,7,8^. These include 2-Cys Prdxs (Prdx1–4) that contain two cysteine residues: a peroxidatic cysteine (C_p_) and a resolving cysteine (C_r_). They form dimers arranged in antiparallel fashion with C_p_ in one monomer facing C_r_ in the other. Upon peroxide binding, C_p_ forms a sulfenic acid intermediate (C_p_-SOH), which then creates an intermolecular disulfide bond with C_r_. This oxidized dimer can then be reduced back to its active form by thioredoxin (Trx). Atypical 2-Cys Prdxs (Prdx5) also has C_p_ and C_r_, but differs from the typical 2-Cys Prdxs as its dimers are not antiparallel; instead, it forms an intramolecular disulfide bond between C_p_ and C_r_ within the same molecule. Reduction of this bond is also carried out by Trx. The 3rd class of Prdx is 1-Cys Prdx (Prdx6): this Prdx has only one cysteine (C_p_) and lacks C_r_. It forms disulfide bonds within the same molecule and is reduced by π glutathione S-transferase (π-GST), rather than Trx ^9,10^.

Prdx6 is called a “moonlighting protein” with both GSH peroxidase and calcium^2+^-independent phospholipase A_2_ activity (aiPLA_2_) ^11,12^. In contrast to other Prdxs, Prdx6 has two functionally independent active sites: one for peroxidase activity (involving Cys47, His39, and Arg132) and another for its aiPLA_2_ activity (involving His26, Ser32, and Asp140). This separation of activities means that loss of function in one site does not impact the function of the other. The Cys47 residue at active site of Prx6 forms a sulfenic acid intermediate during peroxide reduction, with His39 stabilizing the intermediate and assisting in proton transfer. In contrast, aiPLA_2_ activity is generated by Ser32 as the nucleophile that attacks the ester bond in phospholipids, His26 as the base that facilitates the nucleophilic attack, and Asp140 to stabilize the transition state and to support the hydrolysis reaction. Both activities are pH dependent, i.e., at neutral (pH 7.4), Prdx6 exhibits optimal peroxidase activity while at low pH (pH 4.0), it exhibits maximal aiPLA_2_ activity ^13–15^. This pH dependent switch in activity is believed to involve formation of different oligomeric species (monomer or higher oligomer in case of peroxidase and aiPLA_2_ activity, respectively).

**Figure 1:**
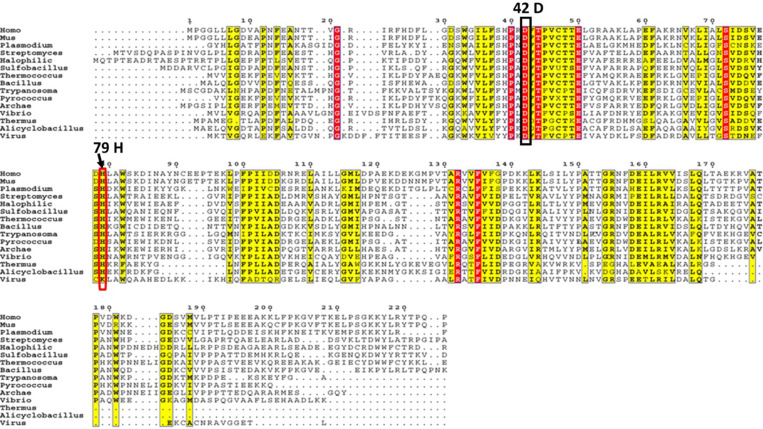
Primary structure of 1-Cys Prdx from different species. Consensus sequences from NCBI aligned using T-Coffee Multiple Sequence Alignment of EMBL-EBI. The red and yellow shading indicates regions of fully-conserved and highly-conserved regions respectively among the species. Residue numbering follows the residues of Prdx6. Black vertical box shows the fully conserved Asp residue. Red box represent the highly conserved His residue throughout the species.

To date, there has been no molecular level understanding of how changes in the pH can help to regulate the peroxidase or aiPLA_2_ functions in Prdx6. It has been shown that Prdx6 undergoes pH dependent oligomerization, i.e., at neutral pH it exists as monomer while it forms dimer or higher oligomers and gain aiPLA_2_ activity at low pH ^16^. However, to date, the role of pH in modulating the oligomeric status of Prdx6 has not been explored sufficiently. It has previously been reported that in case of Prdx1, His113 is protonated at low pH and forms electrostatic interaction with Asp76 ^17^. Formation of this electrostatic pair results in the generation of high order oligomeric species that help to gain other additional functions including chaperone activity. Indeed, similar to Prdx1, there is existence of well conserved His79 and Asp42 in the Prdx6 (Fig. 1). Therefore, we postulated that protonation or deprotonation of certain Asp or His residues might be responsible for the oligomerization of Prdx6 leading to the acquisition of aiPLA_2_ activity. This has lead us to investigate the roles of His79 and Asp42 on the aiPLA_2_ function and oligomerization status of Prdx6. Using two different mutants, His79-Ala and Asp42-Ala, we systematically investigated the role of both His and Asp by analysing the oligomeric status and characterizing the functional activity of the proteins. We discovered that both His and Asp are responsible for the formation of higher oligomers at pH 4.0 and hence in acquiring the aiPLA_2_ function in Prdx6.

## Results

### Mutant Prdx6s (D42A and H79A) exhibit Peroxidase activity but not aiPLA _2_ activity.

Wild type (WT) Prdx6 has been known to exhibit pH-dependent activity i.e., peroxidase activity at neutral pH while aiPLa_2_ at low pH ^14,18–20^. Therefore, we investigated if the mutant Prdx6s possess respective activities. Overnight incubation with excessive amount of DTT was followed by its removal via dialysis against the standard buffer (pH 7.4). The thiol content of the recombinant WT Prdx6 and its mutant Asp42A (D42A) and His79A (H79A) Prdx6s was measured using DTNB assay^21^ to check the oxidation state of Prdx6’s. More than 90% of the Prdx6 remained in the reduced state for 6 h after DTT removal (see supplementary Table 1).

**Figure 2:**
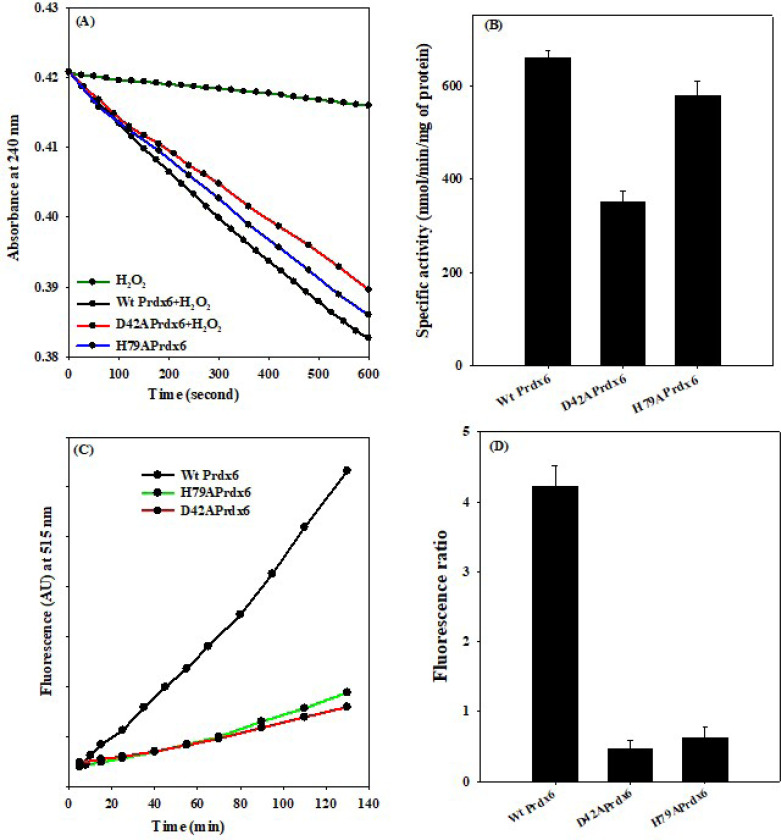
**(A)**. Determination of **Peroxidase activity** of Prdx6 and its mutant, D42A and H79A using H_2_O_2_ decay assay as the extent of consumption of H_2_O_2_ upon reacting with Prdx6 is monitored by the decrease in absorbance at 240 nm (ε_240_ for H_2_O_2_ being 43.6 M^−1^cm^−1^). **(B)** The enzymatic activity estimated for Prdx6 and its mutant is expressed as nmol of reduced H_2_O_2_ / min. mg of Prdx6. Estimation of calcium independent **Phospholipase activity (aiPLA_2_)** of Prdx6 and its mutant as fluorescence emission plot (against time) (C) of EnzChek^®^ Phospholipase A_2_ substrate incorporated in liposomes (prepared by mixing of 30 μl 10 mM Dioleoylphosphatidylcholine, 30 μl 10 mM Dioleoylphosphatidylglycerol, and 30 μl1 mM PLA2 substrate) with addition of Prdx6 at temperature, 25 °C and (D) its fluorescence ratio (515/575).

The time-dependent Prdx6 mediated hydrolysis of H_2_O_2_ (shown in Fig. 2A) shows that H_2_O_2_ exhibits self-degradation potential in the absence of the enzymes and the rate of this degradation is enhanced significantly upon addition of the WT Prdx6. Mutant Prdx6s also exhibit this hydrolytic potential towards H_2_O_2_, albeit at a slower rate than that of WT Prdx6 (Fig. 2B and Table 1). The peroxidase activity of Prdx6 and its mutants calculated as 2nd order rate constant of Prdx6 was also determined using HRP competitive assay^22^ (supplementary Fig. 1). The activity parameters measured using the competitive assay are independent of variability in Prdx6 turnover rate due to varied concentrations of Prdx6. The estimated values of specific activity and 2nd order rate constant of Prdx6 (Table 1) agree well with earlier reports ^23^.

In terms of aiPLA_2_ functions, there is large difference between the time-dependent binding affinity of the phospholipid with the WT and mutant Prdx6s. At the end of the 2 hrs incubation, WT Prdx6 exhibit a relative fluorescence intensity of 5 while the mutant proteins exhibit only around 1.5 (Fig. 2C). The distinct fluorescence resonance energy transfer (FRET) emission of WT Prdx6 at 515/575 nm shows higher fluorescence ratio at pH 4.0 (Fig. 2D and Table 2) consistent with previous reports but this is minimal for the mutant. The results indicate that catalytically, the mutant proteins fail to exhibit aiPLA_2_ activity but exhibits only the peroxidase function and show that the formation of electrostatic interaction at pH 4.0 is required for aiPLA_2_ activity.

### WT Prdx6 forms higher oligomer while mutant Prdxs are dimeric.

**Figure 3:**
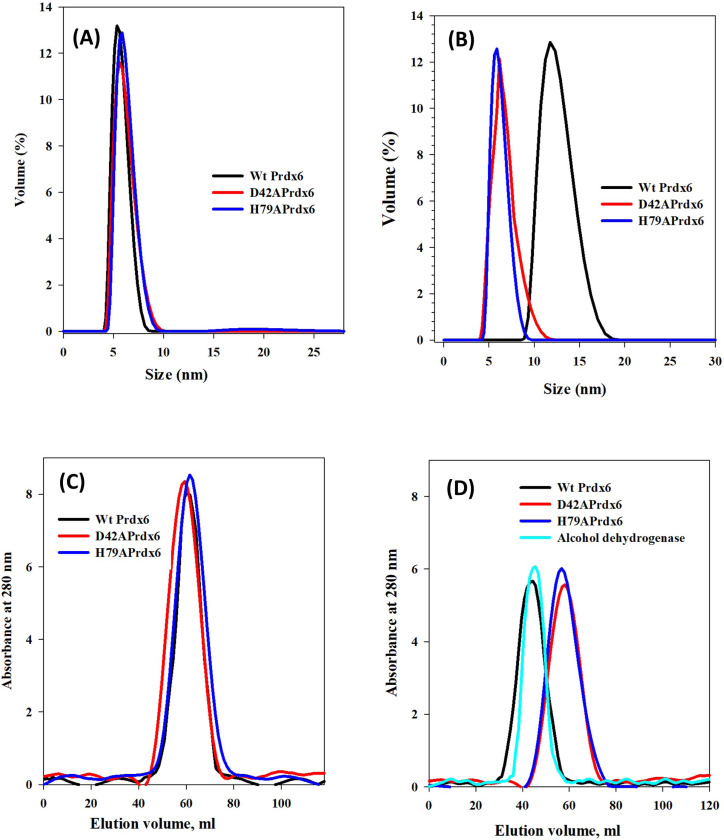
**Dynamic light scattering (DLS)**measurement as the size distribution by volume of Prdx6 and its mutant **D42A and H79A** at **(A)** pH 4 and **(B)** pH 7.4. Measurement of elution volume of Prdx6 and its mutant as absorbance at 280 nm through Size Exclusion Chromatography at pH 4 **(C)** and 7.4 (D).

To probe for the oligomeric status of the WT and mutant Prdxs, we have measured the hydrodynamic diameter $\left(H_{\text{d}}\right)$ of the proteins at both pH 7.4 and 4.0. Raw data representing the size vs volume reaction are shown in Fig. 3A and B and the estimated hydrodynamic diameter along with the percent volume fractions is shown in Table 3. WT Prdx6 remain dimeric with hydrodynamic size around 5 nm, while, upon transfer to low pH medium, higher oligomers are formed with hydrodynamic size almost double to that of the WT. On the other hand, mutant Prdx6s remain dimeric at both the neutral and low pH with hydrodynamic size ranging from 5.5–5.8 as shown (Table 3).

Size exclusion column (SEC) of the WT and mutant proteins depicts the collected elution profiles of the WT and mutant proteins at pH 7.4 and 4.0 (Fig. 3, C and D). The apparent molecular weight of Prdx6 at pH 7.4 and 4.0 using the calibration curve from the elution volume obtained after passing through SEC were determined under different physiological pH (Table 4). Both WT and mutant proteins exhibit similar elution profiles at neutral pH but not at low pH. At low pH, WT Prdx6 elutes as dimer and becomes a higher oligomer with an elution volume that is nearly three times greater than that at pH 7.4. The oligomeric form of WT Prdx6 at pH 4.0 has an apparent molecular weight of approximately 158 kDa, which is consistent with the elution profile of yeast alcohol dehydrogenase (150 kDa) (Fig. 3D). However, for the mutant proteins, the elution profiles at pH 7.4 and 4.0 almost are identical. The oligomeric states of WtPrdx6 and its mutants, D42A and H79A, were also investigated under varying pH conditions using native-PAGE as shown in supplementary Fig. 2. The proteins were dialyzed against the standard buffer at pH 7.4 and 4.0, and then were electrophoresed in a continuous 7.5% PAGE under non-reducing and non-denaturing conditions in presence of two markers, bovine serum albumin (BSA, Mr 66.5 kDa, pI 4.7–4.9) and high molecular weight horse ferritin (Mr 450 kDa, pI 4.4). WtPrdx6, D42APrdx6 and H79APrdx6 at neutral pH appears as dimer (∼50 kD), comparable in size to monomeric BSA (Mr 66 kD). However, both D42APrdx6 and H79APrdx6 at low pH exhibit bands near the size of the dimer as BSA (supplementary Fig. 2A). But WtPrdx6 at low pH 4.0 migrates a shorter distance that is significantly lagging behind of both D42APrdx6 and H79APrdx6, as shown in supplementary Fig. 2B. These observations confirm that WtPrdx6 and its mutant exists as different oligomeric states at both pH 7.4 and 4.0.

### Wt and mutant Prdx6 exhibit different thermodynamic stabilities.

**Figure 4:**
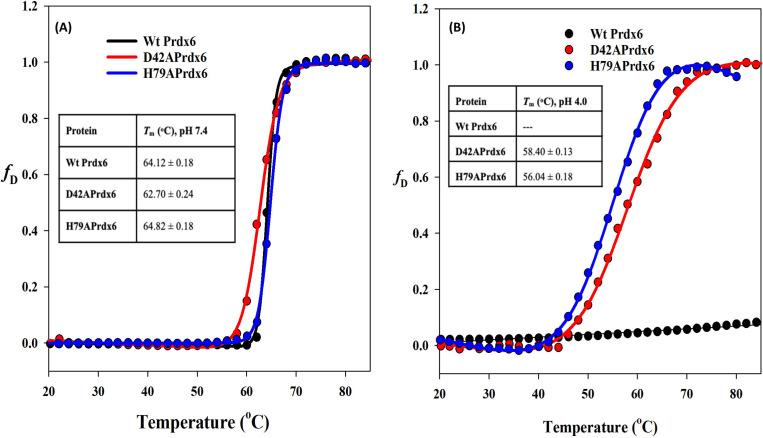
**Thermal-induced denaturation of Prdx6 and its mutant D42A and H79A** as recorded following changes in [*q*]_220_ from 20°C to 80°C at a rate of 1°C/min using Circular Dichroism spectroscopy at pH 7.4 (A) and 4.0 (B).

We also measured the thermodynamic stability of both the mutant and Wt Prdx6s by evaluating heat-induced denaturation of the proteins at both pH 7.4 and 4.0. Representative heat-induced denaturation profiles and the estimated $T_{\text{m}}$ and $\text{Δ}H_{\text{m}}$ are shown in Fig. 4. We observed no significant difference between the $T_{\text{m}}\text{s}$ of the WT and mutant proteins at neutral pH. However, at low pH, WT Prdx6 could not undergo any transition within the measurable temperature range. In contrast, the mutant proteins could exhibit complete transitions with defined pre- and post-transition baselines and their $T_{\text{m}}\text{s}$ that were nearly identical to that of the WT.

### Structural studies of WT and mutant Prdx6s

**Figure 5:**
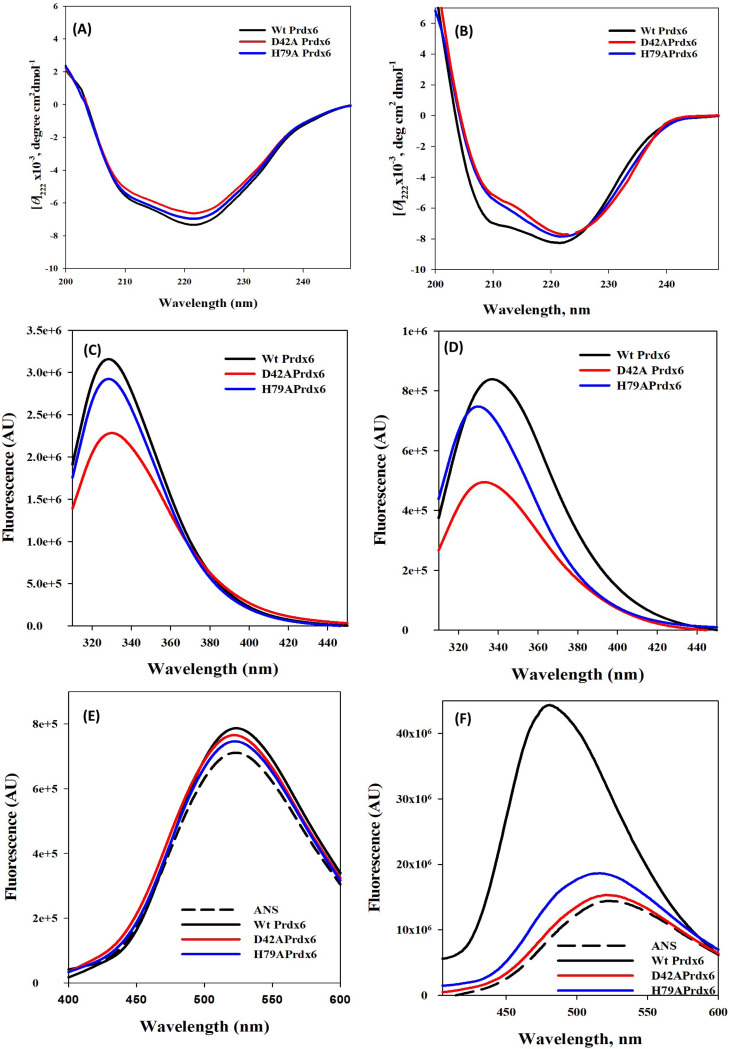
**Conformational and thermodynamic measurement of** Prdx6 and its mutant, **D42A and H79A**. Far-UV CD measurements at pH 4.0 (A) and pH 7.4 (B). Tryptophan fluorescence at pH 4.0 (C) and pH 7.4 (D); and ANS measurements at pH 4.0 (E) and pH 7.4 (F) of Prdx6 and its mutant were measured at standard buffer Na-Acetate buffer, pH 4.0 **and** 50mM Tris-HCl, 100mM NaCl, pH 7.4 at 25°C at 25°C. All spectra are mean of three independent experiments.

We performed structural studies of the WT and mutant proteins by using various spectroscopic techniques so as to gain molecular insight for the formation of species of higher oligomers by WT but not by the mutant proteins at low pH. Far-UV CD of Prdx6 and its mutant observed at pH 7.4 from the Fig. 5A shows that there is no any apparent change on the secondary structure content of WT Prdx6 and its mutants. This is confirmed by the content of alpha-helix content obtained by measuring MRE at $\theta_{222}$ (supplementary Table 2). We also monitored the effect of pH on the secondary structure of the proteins WT Prdx6 and its mutant under low pH as shown in Fig. 5B. Far-UV CD measurements reveal that there is apparent change on the secondary structural contents of the proteins as the spectral properties of the mutants show decrease MRE around $\theta_{208}$ as compare to the WT Prdx6.

The tryptophan fluorescence was scanned in the wavelength 310–450 nm after excitation at 295 nm to evaluate the effect of mutation of Prdx6 in the local environment of tryptophan (Trp) residues of Prdx6. The mutant Prdx6 shows decrease in fluorescence in comparison to wild type Prdx6 at pH 7.4 (Fig. 5C). We also measured the tryptophan fluorescence of WT Prdx6 and its mutant at pH 4.0 (Fig. 5D). Although the mutant Prdx6 shows decrease in fluorescence, the Trp micro-environment have been blue shifted relative to the WT Prdx6. Thus, the Trp chromophores are apparently shifted to the more non polar environments that may help to increase the structural integrity of the mutants as a consequence of the mutations. Such conformational reshuffling can bring about large alterations in the internal packing of the proteins resulting in the exposure vs burial of many of the hydrophobic groups to the solvent.

ANS fluorescence measurements of WT Prdx6 and its mutants D42A and H79A at neutral pH have evaluated to detect accessible hydrophobic surfaces on proteins (Fig. 5E). There is no increase in the ANS fluorescence of the proteins at the neutral pH indicating that ANS cdoes not bind to Prdx6 after mutation. However, WT Prdx6 exhibits a large degree of ANS binding as exemplified by blue shift and increase in fluorescence relative to ANS alone (control) at low pH (Fig. 5F). On the other hand, mutant Prdx6 fails to exhibit binding to ANS as observed by decrease in fluorescence along with no blue shift. These results indicate that higher oligomer formation of the Prdx6 at low pH might involve hydrophobic contacts which is absent in the mutant proteins.

## Discussion

**Figure 6:**
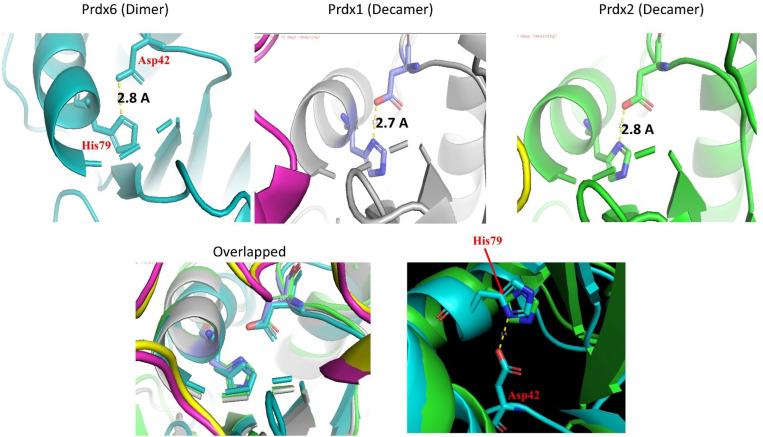
Illustration of structural forms of Asp42 and His79 in different Peroxiredoxin family. The distance between Oxygen atom of Asp42 and Nitrogen atom of His 79 are similar in different Peroxiredoxin family. (A) Prdx6 in dimeric form (represented from PDB ID: 1PRDX) (B) Prdx1 in decameric form (represented from PDB ID: 2Z9S (C) Prdx2 in decameric form (represented from PDB ID: 7KJ0 (D) Overlapped view of Prdx6, Prdx1 and Prdx2. Both the residues of Asp42 and His79 of Prdx6 and corresponding residues of Prdx1 and Prdx2 are aligned and have similar conformation. (E) Cartoon showing interaction of H79 with D42 in Prdx6.

Prdx6 and functionally related proteins essentially exhibit pH dependent activities by changing the oligomeric status. However, it is not yet known how a low pH induces the oligomerization process in these proteins. Speculatively, previous studies have reported the pH induced formation of an electrostatic interaction due to protonation of His113 is primarily responsible for the formation of high order oligomers in Prdx1 ^17^. Since, Prdx6 has also been known to generate dimers or other higher oligomeric species to gain of aiPLA_2_ function at low pH values, we investigated the necessity for formation of such electrostatic interaction in the process. Prdx6 does not have a conserve His or Asp residues at 113 or 76 positions (Fig. 1). However, a well conserved His and Asp residues are present at the position 79 and 42 respectively. The Asp42 and His79 residues of Prdx6 align with corresponding residues in Prdx1 and Prdx2 and exhibit a similar conformation based on the overlapping of their crystal structures. The distance between the oxygen atom of conserved Asp and the nitrogen atom of conserved His is consistent across different members of the Prdxs family (illustrated in Fig. 6). Such distance between Asp and His is appropriate for the formation of electrostatic interaction once His is protonated. Furthermore, theoretical pKa predictions of the His79 and Asp42 side chains in the crystal structure of Prdx6, using the PROPKA 3.0 program, are 8.18–8.40 and 1.21–1.80, respectively ^24^. At the low pH, where there is maximimum aiPLA_2_ activity, His79 remains protonated while Asp42 is negatively charged. This charge difference can result in electrostatic interactions between the different subunits of Prdx6, promoting its oligomerization. Thus, we investigated if Asp42 and His79 residues are (i) essential for oligomerization and (ii) responsible for aiPLA_2_ function.

In the present study, we investigated the hypothesis that the formation of electrostatic interaction driven by the protonation of His79 and Asp42 because of lowering pH is necessary for the oligomerization process of Prdx6. Thus, we have intentionally generated two different mutants by substituting His79 by Ala or Asp42 by Ala. We presumed that if the proteins fail to form the pH driven electrostatic interaction, oligomer formation would not be initiated. Our results on the measurements of hydrodynamic radius, size exclusion chromatography and native-PAGE indicate that change in pH does not influence the nature of oligomeric status of the mutant proteins as compared with the behaviour of WT Prdx6. Interestingly, loss of oligomer formation at low pH corresponds well with the loss of the aiPLA_2_ function of Prdx6. Mutations do not largely affect the peroxidase function of the proteins. Therefore, the results are similar to the previous report on the behaviour of Prdx1. It is also worth noting that the catalytic pocket of the peroxidatic site comprises 3 important residues, Cys47, His39, and Arg132, which are far away from the conserved sites of Asp42 and His79 and do not impact the catalytic core after substitutions by Ala.

It has also been reported previously that such low pH induced oligomeric Prdx6 is highly stable and thermotolerant ^16^. Therefore, it was also important to verify, if the mutant Prdx6 also exhibit thermotolerance at pH 4.0. We investigated this possibility with heat-induced denaturation of the WT and mutant Prdx6 at both pH 7.4 and 4.0. As expected, mutant proteins at pH 4.0 were found to undergo thermal melting and the $T_{\text{m}}$ values are nearly identical to that of the WT Prdx6 (at pH 7.4). The results further led us to believe that the formation of low pH driven electrostatic interaction might be in part responsible for the thermotolerance of WT Prdx6. Thus, the electrostatic interaction is not only required for the oligomer generation but also for the gain thermotolerance.

We further investigated the structural consequences of the mutations at both the neutral and low pH values. It is evident in Fig. 5A that at neutral pH, there is not much significant difference in the secondary structure of the mutant proteins relative to the WT Prdx6 at neutral pH. However, there is apparent alterations in the micro-environments of the Trp residues of the mutant proteins as revealed by the observed decrease in Trp fluorescence as compared to the WT fluorescence. Such alterations in the Trp microenvironment of the mutant proteins do not appear to affect the overall packing of the hydrophobic groups as the ANS binding behaviour is almost identical between the WT and mutant proteins. Thus, there is little structural consequences to the mutations at neutral pH.

In contrast to the relatively small structural changes of the mutant Prdx6 that are observed at neutral pH, relatively large changes in terms of the secondary structural content of the proteins are found at low pH. Indeed, the alpha helical content at neutral pH is decreased by around 20% relative to the WT Prdx6 (Fig. 5B). As a result, there occur concomitant changes in the microenvironment of the Trp residues relative to the WT. However, there is no significant binding of ANS to the mutant proteins indicating that solvent exposed hydrophobic clusters at the protein surface does not exist. On the other hand, WT Prdx6 exhibits large binding behaviour of ANS suggesting the presence of large number of solvent exposed hydrophobic groups. Thus, it is imperative that formation of electrostatic contact results in large structural consequences leading to the exposition of hydrophobic clusters that might favour the assembly of Prdx6 with higher oligomer. On the other hand, in the mutant forms, in the absence of the electrostatic interaction, the native protein cannot initiate sufficient structural rearrangement that is required for oligomer formation. Thus, it is confirmed that formation of electrostatic interaction results in large structural reshufling that involves the exposure of hydrophobic groups and hence favor the assembly of hydrophobic contacts between two Prdx6 monomers. In support, it has been reported that hydrophobic residues (Leu 145, Ile 147, Leu 148 and Tyr 149) between β-strands (β7 strands from each monomer) may play an important role in stabilizing the protein dimer ^25^. Additionally, the hydrophobic amino acids L145 and L148 play an important role in homodimerization of Prdx6 as well as in its heterodimerization with πGST ^26^.

There is no concrete explanation of how His79 and Asp42 exclusive interaction or its accompanying micro-environment induces oligomer formation in Wt Prdx6. However, it can be explained that in Prdx6, at a pH of 4.0, His79 becomes protonated while Asp42 retains its negative charge, resulting in electrostatic interactions between these residues. Structure of Prdx6 formed at pH 4.0 is very complex not a conventional type as the protein gains thermotolerance at this condition (see Fig. 4). Further examination of the local residues and structures in and around the residues revealed that Asp42 is located within the loop that connects β-strand 3 to α-helix 2 (α2) (where His79 is present). It has been already reported that this loop plays a critical role in peroxidase activity and A-type dimerization, where α-helices from two monomers interact and form dimer. Additionally, glutathionylation of a cysteine residue in α-helix 2 has been shown to promote helix unwinding and disassembly of the homodimer ^27^. Therefore, it is possible that formation of His79-Asp42 electrostatic interaction impact the position of the loop that helps in repositioning the orientation of the helices in one monomer so that it could interact favorably with another monomer to form dimer.

Since protein folding or unfolding is a cooperative process wherein all forces stabilizing the native structure act in a concerted manner, acquision of an electrostatic interaction at pH 4.0 in the Wt enzyme might help to facilitate formation of other additional cooperative bonds/forces bringing about accompanying structural alterations exemplified by the exposition of hydrophobic groups in the monomers leading to oligomer formation. Thus, formation of electrostatic interaction is not the exclusive event toward the whole structural consequences.

## Conclusion

We are sure that formation of electrostatic interaction between His79 and Asp42 is responsible for the gain of aiPLA_2_ function of Prdx6. Such a formation of electrostatic interaction also helps not only in oligomer formation essential for aiPLA_2_ activity but also in gaining thermotolerant conformation in Prdx6 at pH 4.0. On structural basis, the formation of electrostatic interaction results in large structural reshufling including the exposition of hydrophobic groups that eventually helps in the thermodynamically favorable hydrophobic contact formation. From the thermodynamic perspectives, at low pH, native protein is expected to experience conformational tension as a result of the two opposing forces (repulsive forces contributed by positive charges and electrostatic interaction between His79-Asp42). Eventually, such conformational reshuffling results in the exposition of hydrophobic clusters in the monomeric protein which is thermodynamically unfavorable. However, this is followed by a hydrophobic contact formation between two monomers leading to the assembly of the two monomers making the thermodynamically unfavorable process favorable in the dimer.

## Materials and Methods

Isopropyl β-d-1-thiogalactopyranoside (IPTG), Horse peroxidase, DTT (DL-Dithiothreitol), yeast alcohol dehydrogenase, Sodium acetate, analytical grade Tris-HCl, bovine serum albumin (BSA), Ferritin and 8-Anilinonaphthalene-1-sulfonate (ANS) were purchased from Sigma-Aldrich (St. Louis, MO, U.S.A.). Amicon protein concentrator having 10 kD cut off were purchased from Millipore Corporation (Burlington, Massachusetts, United States). Dialysis tubing with a molecular weight cut-off of 6–8 kDa was purchased from Sigma-Aldrich (St. Louis, MO, USA). Plasmid isolation kit was obtained from Qiagen, Hilden, Germany while the EnzChek^®^ Phospholipase A2 Assay Kit, restriction enzymes such as Nde1 and sap1 were from Invitrogen, USA. The pH 4 and pH 7 standard buffer solutions used for calibrating the pH meter at 25°C were purchased from Merck (India). Chitin resin beads were obtained from New England Biolabs (NEB) (Ipswich, MA, USA). Ethylenediaminetetraacetic acid (EDTA) and sodium bicarbonate were sourced from Glaxo Laboratories (India). All the above chemicals were of analytical grade reagents that can be used without further purifications.

## Methods

### Preparation of recombinant protein

Recombinant rat wild-type was cloned between the NdeI and SapI sites of the pTYB1 vector (New England BioLabs), as described earlier ^13,28^. This vector comprises a chitin-binding domain for affinity purification and an intein for precise self-cleavage of the purification tag such that no extra residues remain after purification that could interfere with either the peroxidase or aiPLA_2_ activities of Prdx6. The mutants Asp43Ala and His79Ala mutants of Prdx6 were created using QuikChange^™^ site-directed mutagenesis (Stratagene).

Protein expression was carried out in BL21(DE3) cells, grown overnight at 20°C. Cells were harvested by centrifugation, followed by sonication. The lysate was clarified by additional centrifugation, and the supernatant was subjected to purification using a chitin affinity column as previously described^27^. Subsequent size-exclusion chromatography yielded a homogenous protein product, confirmed by SDS-PAGE (see Supplementary Fig. 3).

### Hydrogen Peroxide Decay Assay

Measuring consumption of hydrogen peroxide in the presence of GSH as an electron dono can be used to assay the glutathione peroxidase activity of Prdx6 ^11,28^. The standard reaction buffer at different pH values were (a) 10 mM Tris-HCl, 0.1 mM EDTA, 2 mM NaN_3_, and 0.2 mM GSH (pH 7.0) and (b) 40 mM Na acetate, 0.1 mM EDTA, 2 mM NaN_3_, and 0.2 mM GSH (pH 4.0). The specific activity of Prdx6 is computed using the equation:

$$\text{Enzyme activity}=\frac{\text{Δ Abs}/\text{min}}{\left[\text{E}\right]}\times 10^{3}$$

where, ΔAbs/min as change in the OD_240_ per minute, and [E] as the concentration of enzyme used in ‘mg/ml’. Enzymatic activity was reprented as nmol of H_2_O_2_ reduced per minute per mg of protein. Data for multiple replications are represented as mean ± S.E.

### Ca independent PLA activity (aiPLA)

Beside its peroxidase activity, Prdx6 exhibits Ca^2+^ independent intracellular phospholipase, aiPLA_2_ activity, that hydrolyze the sn-2 ester linkage of phospholipids maximally at pH 4.0. The PLA_2_ activity of Prdx6 was quantified using the EnzChek^®^ Phospholipase A_2_ Assay Kit by measuring the increase in fluorescence intensity at a single wavelength approximately 515 nm and distinct fluorescence resonance energy transfer (FRET) emission at 515/575 nm after excitation at ~ 460 nm of the substrate prior to and after cleavage in the absence of calcium as described previously^16^ Here we used 0–10 units/mL of PLA_2_ from honey bee venom as positive controls and no PLA_2_ as a negative control. Each reaction was conducted in a total volume of 50 μL, containing 100 μg of purified enzyme in standard buffer- 40 mM Na-acetate buffer, pH 4.0 which was then incubated with an equal volume of a substrate-liposome mixture (prepared by combining 30 μL of 10 mM dioleoyl phosphatidylcholine, 30 μL of 10 mM dioleoylphosphatidylglycerol, and 30 μL of 1 mM PLA_2_ substrate) for 10 minutes at room temperature. .

### Dynamic light scattering measurements

Size distribution of particles present in the protein sample was obtained using a Zetasizer Micro V/ZMV 2000 (Malvern, UK) as decrebed previously^16^. Each sample was measured fifteen times with an acquisition time of 30 seconds at sensitivity of 10%. The data were analysed using Zetasizer software provided by the manufacturer to determine the hydrodynamic diameters of the particles and the volume fraction corresponding to each diameter. The protein concentration was 2 mg/ml and all measurements were carried out at 25°C.

### Gel Permeation Chromatography

Analytical SEC experiments at various pH values were performed using a Hi-Prep 26/60 Sephacryl S-200 HR GL column (GE Healthcare) with a flow rate of 0.5 ml min^− 1^. After purification with a chitin affinity column, rat Prdx6 was dialyzed against the standard buffers at pH 7.4 and pH 4.0. The dialysed protein, after concentration, was loaded onto the pre-equilibrated column with the corresponding standard buffer after filtering the protein using a 0.1 mm pore-sized filter (Millipore, Burlington, MA) and degassed thoroughly. The SEC column was calibrated using low and high molecular weight markers (GE Healthcare). A calibration curve was plotted using the gel phase distribution coefficient $\left(K_{\text{av}}\right)$ versus logarithm of the molecular weight $\left(\text{Log}\;M_{\text{w}}\right)$ of the markers usindg the formulae:

(1) $$K_{\text{av}}=\left({\text{V}}_{\text{e}}-{\text{V}}_{\text{o}}\right)/\left({\text{V}}_{\text{c}}-{\text{V}}_{\text{o}}\right)$$

where ${\text{V}}_{\text{e}}$, elution volume; ${\text{V}}_{\text{o}}$, column void volume (39.26) and ${\text{V}}_{\text{c}}$, geometric column volume (120 ml).

### Native-PAGE

Native-PAGE at neutral pH was performed in the standard buffer i.e., 50 mM Tris-HCl, 100 mM NaCl pH 7.4 with running buffer 35 mM Hepes and 43 mM Imidazole, pH 7.4 at 120 v for 1.30 hr with normal polarity while native-PAGE at low pH was done in 40 mM Na-acetate, pH 4, using running buffer of 30mM beta alanine and 20 mM lactic acid (85–90%), pH 3.8, at a constant voltage (180 V) for 90 minutes with reverse polarity. Protein samples were electrophoresed in a continuous 7.5% polyacrylamide gel under non-reducing and non-denaturing condition using two markers, bovine albumin (Mr 66.5kDa, pI 4.7–4.9) and high molecular weight horse ferritin (Mr 450kDa, pI 4.4). Coomassie brilliant blue G-250 was used for the detection of the proteins on the gel.

### Thermal-Induced Denaturation

Heat-induced denaturation of Prdx6 was monitored using a Jasco-750 CD Spectropolarimeter equipped with a Peltier-type temperature controller as described earlier^27^. The CD measured mean residual ellipticity at $[\theta {]}_{220}$ as a function of temperature is normalized at the start of the temperature scan. As previously demonstrated ^28^, each thermal denaturation curve was analyzred using a two-state unfolding model, assuming a linear temperature dependence for both pre-and post-transition baselines ^14,29^

(2) $$\text{y}(\text{T})=\frac{\left[\left({\text{y}}_{\text{N}}+{\text{m}}_{\text{N}}\text{T}\right)+\left({\text{y}}_{\text{D}}+{\text{m}}_{\text{D}}\text{T}\right)\right]\text{exp}\left[-{\text{ΔH}}_{\text{m}}/\text{R}\left(1/\text{T}-1/{\text{T}}_{\text{m}}\right)\right]}{1+\text{exp}\left[-{\text{ΔH}}_{\text{m}}/\text{R}\left(1/\text{T}-1/{\text{T}}_{\text{m}}\right)\right]}$$

y(T) is the observed mean residue ellipticity at a given temperature, ${\text{m}}_{\text{N}}$ and ${\text{m}}_{\text{D}}$ are slopes of the native and denatured baselines, while ${\text{y}}_{\text{N}}$ and ${\text{y}}_{\text{D}}$ are the intercept of the native and denatured baselines, respectively. T is the temperature in Kelvin, ${\text{T}}_{\text{m}}$ is the melting temperature in Kelvin and ${\text{ΔH}}_{\text{m}}$ is the enthalpy change of denaturation at melting temperature and R is the universal gas constant. Curve fitting of the data points was done in Sigma Plot 13.0 software, from Systat Software, Inc., San Jose, CA, USA using Eq. (2) in the dynamic fit.

### Circular dichroism (CD)

CD measurements were recorded in standard buffers using Jasco-750 CD spectropolarimeter at 25°C as performed earlier^27^. Spectra were recorded in the far-ultraviolet region (190–260 nm), with a bandwidth of 1.0 nm, a step size of 1 nm, an integration time of 30 s, and with three repeats. A fused quartz cell with a pathlength of 0.1 cm was used. The protein concentration in standard buffers at pH 7.4 and 4.0 was 10 μM. The results of all the CD measurements are expressed as mean residual ellipticity $\left({\left[\theta \right]}_{\lambda }\right)$ in deg cm^2^ dmol^− 1^ at a given wavelength λ (nm) using the relationship: ${\left[\theta \right]}_{\lambda }=\theta_{\lambda }{\text{M}}_{\text{o}}/10cl$, where $\theta_{\lambda }$ is the observed ellipticity in millidegrees at wavelength λ, ${\text{M}}_{\text{o}}$ is the mean residual weight of the protein, c is the protein concentration (mg/cm^3^), and l is the path length (cm).The change in the far-UV CD is quantitated by measuring the alpha-helix content using the formula: alpha-helix $(\%)=[\left(-{\left[\theta \right]}_{222}+3000\right)/36000+3000]\times 100$, where ${\left[\theta \right]}_{222}$ is the mean residual ellipticity at 222 nm ^29^.

### Fluorescence spectroscopy

Fluorescence spectroscopy was conducted using a PTI spectrofluorometer (Photon Technology International, Lawrenceville, NJ) with a temperature-controlled water bath sample holder, a single photon counting system, and dual fluorescence and absorbance channels. Measurements were taken at 22°C with 1μM protein in standard buffers, using microquartz fluorescence cuvettes with a 0.3 cm pathlength and 1 nm excitation and emission slits. Tryptophan fluorescence was recorded from 310–450 nm after excitation at 295 nm to exclude tyrosine fluorescence.

ANS fluorescence spectra of Prdx6 were collected from 400 to 600 nm after excitation at 360 nm. For both Trp and ANS fluorescence measurements, the protein concentration was 1 μM, and ANS was used at 16 μM. All the measurements were performed in microquartz fluorescence cuvettes with 0.3 cm pathlength.

### Statistical analysis

Results are expressed as mean ± SD. Data points for thermal-induced denaturation were curve-fitted using dynamic fit function in Sigma Plot 13 software .

## Supplementary Material

Supplement 1

## Figures and Tables

**Table 1:** Peroxidase Activity of Prdx6 and its mutant D42A and H79A at pH 7.4

Protein	Peroxidase activity (nmol/min/mg of protein)	2nd order constant, *k* (M^− 1^ s^− 1^)
Wt Prdx6	660 ± 30	3.85 × 10^7^
D42APrdx6	540 ± 20	2.56 × 10^7^
H79APrdx6	600 ± 20	3.69 × 10^7^

**Table 2:** Phospholipase Activity comparison of Prdx6 and its mutant, D42A and H79A at pH 4.

Protein	Fluorescence ratio (515/575)	Fluorescence at 515 nm (10^4^)
Wt Prdx6	4.23 ± 0.34	632.52
D42APrdx6	0.45 ± 0.12	153.86
H79APrdx6	0.62 ± 0.15	159.32

**Table 3:** Hydrodynamic size of Prdx6 and its mutant, D42A and H79A under different pH 7.4 and 4.0 measured by DLS.

Protein	pH 7.4		pH 4.0	
	Hydrodynamic size, *H*_d_ (nm)	Volume fraction (%)	Hydrodynamic size, *H*_d_ (nm)	Volume fraction (%)
Wt Prdx6	5.35 ± 0.13	98.2	12.30 ± 0.81	98.5
D42APrdx6	5.56 ± 0.21	97.4	5.96 ± 0.26	98.8
H79APrdx6	5.88 ± 0.15	98.6	5.92 ± 0.31	99.2

**Table 4:** Measurement of elution volume and apparent molecular weight of Prdx6 and its mutant, D42A and H79A under different pH 7.4 and pH 4.0 using FPLC

Protein	pH 7.4		pH 4.0	
	Elution volume (ml)	Molecular weight (kD)	Elution volume (ml)	Molecular weight (kD)
**Wt Prdx6**	60.3 ± 0.08	47	43.3 ± 0.09	**158**
**D42APrdx6**	59.2 ± 0.21	51	57.2 ± 0.18	**59**
**H79APrdx6**	61.1 ± 0.05	45	57.1 ± 58	**58**

## Data Availability

All data generated or analysed during this study are included in this published article and its supplementary information files.
