# Supplementary material for: Role of Aspartate 42 and Histidine 79 in the aiPLA2 activity and oligomeric status of Prdx6 at low pH
Source: Res Sq. 2024 Dec 23:rs.3.rs-5129146. Preprint. [Version 1] doi: 10.21203/rs.3.rs-5129146/v1 (PMC11703348; doi:10.21203/rs.3.rs-5129146/v1)
Supplement: Supplement 1 [file NIHPPRS5129146v1-supplement-1.pdf]

Supplementary Files

This is a list of supplementary files associated with this preprint. Click to download.

- Supplementary.docx
